# Effects of fermented *Rosa roxburghii* Tratt pomace on growth performance, lipid metabolism, antioxidant activity, and amino and fatty acid profile in goats

**DOI:** 10.1371/journal.pone.0342308

**Published:** 2026-02-03

**Authors:** Rui Chen, Fu Wang, Shuanglong Xie, Yiming Ban, Chengcheng Gao, Peiyao Li, Jixiao Qin, Yiqing Xu, Qi Lu, Xu Wang, Xingzhou Tian

**Affiliations:** 1 Laboratory of Animal Genetics, Breeding and Reproduction in the Plateau Mountainous Region, Ministry of Education, College of Animal Science, Guizhou University, Guiyang, Guizhou, China; 2 Guizhou Testing Center for Livestock and Poultry Germplasm, Guiyang, Guizhou, China; 3 School of Animal Technology and Innovation, Suranaree University of Technology, Nakhon Ratchasima, Thailand; King Saud University / Zagazig University, EGYPT

## Abstract

*Rosa roxburghii* Tratt pomace, is a juice extraction rich in polyphenols with strong antioxidant activity. The objective of this study was to evaluate the effect of fermented *Rosa roxburghii* Tratt pomace (FRRT) on growth performance, plasma biochemistry and antioxidant status, and *longissimus dorsi* muscle amino- and fatty-acid profiles in meat goats. Twenty-four goats with similar body weights (30.7 ± 4.71 kg) were allotted in a completely randomized design to either basal diet (CON) or the same diet containing 7% (LF) or14% (HF) of FRRT on a total mixed ration (TMR) dry matter (DM) basis for 60 d following 14 d adaptation. FRRT did not alter dry matter intake or average daily weight gain, but reduced (*P* < 0.05) the feed conversion ratio (FCR) compared to CON. Plasma creatinine and total cholesterol increased (*P* < 0.05) with FRRT, while low-density lipoprotein cholesterol (LDL-C) was numerically higher in the HF group (overall *P* = 0.069). FRRT increased (*P* < 0.05) the plasma total antioxidant capacity and catalase; malondialdehyde tended to be lower (*P* = 0.067) HF group than CON. In muscle, the 14% FRRT diet increased essential, non-essential, flavor and total amino acids (*P* < 0.05), with higher concentrations of several individual amino acids (e.g., threonine, serine, glycine, tyrosine, lysine, arginine). Among fatty acids, C15:0 decreased in 7% FRRT (*P* < 0.05) and arachidonic acid (C20:4n-6) increased (*P* < 0.05), whereas sums of SFA, MUFA and PUFA did not differ. In conclusion, 7–14% FRRT (DM basis) improved feed efficiency and antioxidant status and favorably modified selected muscle amino- and fatty-acid traits, but elevated circulating cholesterol; the lipid responses warrant further study.

## Introduction

With rapid growth of animal husbandry, feed scarcity has become a key constraint [1], especially in China, where raw materials are limited and efficient use of feed resources is a bottleneck [2]. Butylated hydroxyanisole and butylated hydroxytoluene (BHA/BHT) are effective preservatives in meat. [3]. However, safety concerns over synthetic additives have driven interest in plant-derived antioxidants for animal feeding [4].

Polyphenols are a source of natural antioxidant, which widely present in plants and are generally classified as molecules with multiple phenolic hydroxyls [5]. An increasing number of studies have shown that polyphenol compounds are popular among researchers due to they are safe to animals and have no side effects relative to synthetic antioxidants, they act as protective nutritious components in feeds, increase the strength of animal antioxidants, and improve carcass quality [6,7]. Specifically, various studies have shown that some byproducts contain abundant polyphenolic compounds, which could improve meat quality and animal health [8,9]. This was due to dietary addition of polyphenols could inhibit polyunsaturated fatty acids (PUFA) oxidation and improve PUFA content by inhibiting the hydroperoxides and free radicals in meat, and thus improved the meat quality of ruminants [10,11]. For example, Ahmed et al. [12] revealed that the feeding of green tea byproducts increased the proportions of muscle monounsaturated fatty acids (MUFA), PUFA, and n-6 PUFA in growing goats. Thus, dietary supplemented with polyphenol-rich plant might improve meat quality in ruminants [13].

*Rosa roxburghii* Tratt (RRT) is a medicinal and edible plant widely distributed in the mountainous area of Southwest China [14]. The *Rosa roxburghii* Tratt pomace (RRP) is the residual byproduct of *Rosa roxburghii* after juice extraction, accounting for approximately half of the weight of the fresh fruit [15], and is known for its remarkably high vitamin C, superoxide dismutase (SOD), and polyphenol contents, which are high-quality functional feed materials for ruminants [16]. For example, Song et al. [17] reported that dietary supplementation with RRP did not affect production performance, but it improved plasma protein utilisation efficiency in cattle. Li et al. [18] reported that there were no negative impacts on growth performance, and adding up to 30% *Rosa roxburghii* Tratt residue allowed for feed cost savings without affecting the characteristics of rumen fermentation in Hu sheep. However, RRP has a high moisture content, does not store well, is prone to mould and deterioration, and is often discarded during production, resulting in waste [19]. Therefore, the development of ensiling from RRP has become a trend in the feed industry [20]. Recent research has demonstrated that fermentation can preserve or even increase phenolic content and improve flavor, supporting its use as an animal feed ingredient [21]. In addition, the total phenolic and flavonoid contents of *Rosa roxburghii* Tratt juice increased during fermentation [22]. Thus, the nutritional value of RRP after fermentation is preserved to a large extent, and it is also promising as an animal feed.

Most ruminant studies have used dried RRP, which adds cost and may reduce bioactive components [22]. In contrast, fermentation can preserve traditional nutrients and bioactive compounds, potentially improving bioavailability and animal performance. However, the reports about the effects of fermented *Rosa roxburghii* Tratt pomace (FRRT) on antioxidant potential and meat quality are rare in meat goats. It is hypothesised that FRRT is rich in polyphenol compounds and could improve the blood antioxidant status and meat quality of the *longissimus dorsi* muscle in goats. Accordingly, the aim of this study was to assess the effects of FRRT on growth performance, lipid metabolism, antioxidant activity, and amino acid (AA) and fatty acid (FA) contents in meat goats.

## Materials and methods

### Ethical approval

The study was approved by the Guizhou University (Guiyang, China) Subcommittee of Experimental Animal Ethics (approved number EAE-GZU-2024-E026), dated July 9, 2024. The slaughter experiment was carried out in Guizhou Province Goat Breeding Testing Station Farm. At the end of the feeding trial, three goats randomly selected from each group were stunned by professional slaughterers and slaughtered by bloodletting from the jugular vein.

### Preparation of fermented *Rosa roxburghii* Tratt pomace

The RRP used in this study was obtained from Guizhou Kingvibo Biotechnology Co. Ltd. (Dafang, China). Notably, the excessively high moisture level of fresh RRP makes it unsuitable for fermentation. Therefore, fresh RRP was mixed with wheat bran at 80:20 (fresh basis) to improve fermentability. The RRP–wheat bran mixture was tightly packed into 200 L plastic silage tanks (Zhenjiang Yunxu Plastic Container Technology Co., Ltd., Zhenjiang, China) to exclude air as much as possible, sealed with airtight lids, and fermented for 60 days under ambient temperature conditions (approximately 15–25°C). At opening, the absence of spoilage bacteria (such as mold, odor, etc.) was confirmed by the naked eye and smell. The resulting material is hereafter referred to as FRRT. The polyphenol profile of FRRT (DM basis) is presented in Table 1.

**Table 1:** Polyphenol compounds of pure fermented *rosa roxburghii* tratt pomace.

Item	Polyphenol compounds (ng/mg of dry matter)
Gallic acid	44.7 ± 5.03
3,4-Dihydroxybenzoic acid	10.8 ± 1.41
Protocatechualdehyde	0.83 ± 0.09
4-Hydroxybenzoic acid	5.02 ± 0.32
Phthalic acid	2.25 ± 0.18
Catechin	7.55 ± 0.63
Vanillic acid	8.58 ± 0.44
Caffeic acid	4.22 ± 0.81
Syringic acid	4.46 ± 0.36
Dihydromyricetin	0.08 ± 0.02
Vanillin	1.42 ± 0.09
p-Hydroxycinnamic Acid	6.46 ± 0.39
Syringaldehyde	0.63 ± 0.06
Rutin	0.12 ± 0.02
Vitexin	0.07 ± 0.01
Trans-Ferulic acid	20.3 ± 2.21
Salicylic acid	0.95 ± 0.03
Sinapic Acid	0.62 ± 0.04
Quercetin 3-β-D-glucoside	0.52 ± 0.11
(+)-Dihydroquercetin	2.41 ± 0.07
Genistin	0.08 ± 0.02
Benzoic acid	3.38 ± 0.20
Kaempferol-3-O-glucoside	1.62 ± 0.11
(+)-Dihydrokaempferol	0.89 ± 0.02
Resveratrol	0.05 ± 0.00
Luteolin	0.24 ± 0.01
Quercetin	24.0 ± 1.48
Hydrocinnamic acid	0.96 ± 0.11
Trans-Cinnamic acid	0.99 ± 0.10
Phloretin	0.01 ± 0.00
Apigenin	0.06 ± 0.00
Naringenin	0.87 ± 0.05
Kaempferol	11.3 ± 1.06
Isorhamnetin	0.44 ± 0.02
Gossypol	0.74 ± 0.12

Values represent the means of six replicates (n = 6). mean ± standard deviation.

### Experimental design

Before the feeding trial, a pre-experiment research showed that dietary supplemented with more than 14% FRRT based on the dry matter (DM) of the total mixed ration (TMR) reduced the feeding speed and DM intake (DMI) in goat. Thus, the addition of more than 14% FRRT based on the DM of the TMR might negatively affect the palatability of meat goats. Thus, the feeding experiment was carried out at the Guizhou Province Goat Breeding Testing Station Farm (106.17 E, 26.35 N, Anshun, China). A total of 24 Guizhou black male goats with similar body weights (30.7 ± 4.71 kg) were randomly divided into three treatment groups via a completely randomized design, with 8 replications in each treatment, and 1 goat per replication (*n* = 8). All experimental goats were kept in individual clean pens (1.8 m × 1.0 m). Before the feeding trial, all experimental animals were treated for parasites with a single oral dose of albendazole (10 mg/kg body weight) and a topical application of ivermectin (0.2 mg/kg body weight) to control gastrointestinal and external parasites, respectively. The three treatments were as follows: (1) the control group (CON) goats were fed a basal diet (without FRRT addition), (2) the treatment 1 (LF) goats were fed 7% FRRT based on the DM of the TMR, and (3) the treatment 2 (HF) goats were fed 14% FRRT based on the DM of the TMR. The experimental feeding period was carried out for 74 d, which included a 14-d adaptation period and a 60-d experimental period. The FRRT supplementation levels (7% and 14%) were calculated on a DM basis, and the FRRT was utilized as a roughage feedstuff in the TMR. Thus, the FRRT was first thoroughly combined with roughage in accordance with the addition ratio, and then mixed well with the concentrate to prepare individual experimental TMR diets. All the experimental goats were fed at 9:00 and 16:00 every day. All experimental goats were offered feed and water *ad libitum* throughout the feeding period. The rations were formulated to meet the nutrient requirements recommended by the Chinese standard NY/T 816–2021 [23]. The ingredients and chemical composition of the experimental diets are shown in Table 2.

**Table 2:** The ingredients and chemical composition of experimental diets (dry matter basis).

Item	CON	LF	HF
Oat grass	53.0	46.0	39.0
Hybrid giant napier	7.00	7.00	7.00
Fermented *rosa roxburghii* tratt pomace	0.00	7.00	14.0
Corn	21.4	22.2	23.1
Soybean meal	14.2	13.3	12.4
Salt	0.50	0.50	0.50
Sodium bicarbonate	0.60	0.60	0.60
Compound-premix^*^	3.40	3.40	3.40
Chemical composition, %			
DM	90.0	90.2	89.7
CP	14.5	14.5	14.6
EE	3.94	3.58	3.23
NDF	59.2	55.6	51.7
ADF	31.8	30.5	29.7
Ash	7.70	7.09	6.62

Values represent the means of three replicates (*n* = 3). DM: dry matter; CP: crude protein; EE: ether extract; NDF: neutral detergent fiber; ADF: acid detergent fiber.

* The compound-premix contains a vitamin and mineral premix with the following composition per kg: vitamin A, 150 KIU; vitamin D3 80 KIU, vitamin E, 500 IU, Cu 300 mg, Fe 1500 mg, Zn 1500 mg, Mn 1500 mg, I 30 mg, Co 10 mg, Se 5 mg.

CON: goats were fed a basal diet; LF: goats were fed a basal diet supplemented with 7% fermented *rosa roxburghii* tratt pomace; HF: goats were fed a basal diet supplemented with 14% fermented *rosa roxburghii* tratt pomace.

### Chemical composition

The feed was dried in an oven at 105°C (TNX140030; Shanghai Shinbae Industrial Co., Ltd., Shanghai, China) until a constant weight was achieved to determine DM content (AOAC 934.01). In addition, crude protein (CP; AOAC 988.05; CP content was calculated as N × 6.25), ether extract (EE; AOAC 2003.05), and ash (AOAC 942.05) contents were analysed according to AOAC methods [24]. Neutral detergent fiber (NDF) and acid detergent fiber (ADF) contents were analysed with the use of α-amylase and are expressed as residual ash according to the Van Soest et al. [25].

### Polyphenol compounds

Approximately 0.5 g of pure FRRT sample was weighed, added to a 1.5 mL centrifuge tube, and 0.5 mL of 80% methanol aqueous was added. The mixture was vortexed and subjected to ultrasonic extraction for 30 min at room temperature. Next, the mixture was centrifuged at 12000 × g for 10 min at 4°C, after which the supernatant was harvested for further polyphenol compound analysis. Ultra-high-performance liquid chromatography (Vanquish, UPLC, Thermo, USA) and high-resolution mass spectrometry (MS; Q Exactive, Thermo, USA) were used to analyse the polyphenolic compounds. The UPLC conditions were as follows: the chromatography column was an HSS T3 (50 × 2.1 mm, 1.8 μm); mobile phase A was an ultrapure water solution (containing 0.1% formic acid); mobile phase B was an acetonitrile solution (containing 0.1% formic acid); the flow rate was 0.3 mL/min; the column temperature was 40°C; the injection volume was 2 μL; and the gradient program was 90:10 V/V at 0 min, 90:10 V/V at 2.0 min, 40:60 V/V at 6.0 min, 40:60 V/V at 9.0 min, 90:10 V/V at 9.1 min, and 90:10 V/V at 12.0 min. The MS conditions were as follows: electric spray ion source; sheath gas, 40 arb; auxiliary gas, 10 arb; ion spray voltage, −2800 V; temperature, 350°C; and ion transfer tube temperature, 320°C. The scanning mode was full scan-ddMS2, and the scanning method was negative ion mode. The scanning range of primary mass spectrometry (scan m/z range) was 100–900. A standard curve was established for quantitative determination, and individual polyphenol compounds were calculated from the peak areas of the chromatograms.

### Growth performance

The feed intake and residual feed of each goat were recorded daily during the formal period to calculate DMI. The goats were weighed before the morning feeding on the first day (1 d) and the last day (60 d) of the formal period, and the average daily gain (ADG), body weight change (BWC), and feed conversion ratio (FCR) were calculated by the following formulas: BWC (kg) = final weight (kg) – initial weight (kg); ADG (g/d) = BWC (kg) × 1000/number of feeding days; FCR = DMI (g/d)/ADG (g/d).

### Plasma lipid metabolism and antioxidant activity

On the last day of the experimental period, approximately 10 mL of blood was collected from each goat via the jugular vein using an EDTAK2 negative pressure vacuum blood collection tube (Shijiazhuang Kangweishi Medical Equipment Co., Ltd., Shijiazhuang, China). All blood samples were centrifuged at 4,000 × g for 10 min at 4°C to obtain plasma. The plasma was then immediately aliquoted into sterile cryotubes to avoid repeated freeze-thaw cycles and stored at −80°C until analysis. Each aliquot was thawed only once for a specific assay. All lipid metabolism parameters and antioxidant activities were detected using the commercial reagent kits obtained from Nanjing Jiancheng Bioengineering Institute in China. All the kits in the experiment were validated by the manufacturer and could be used to determine plasma and all procedures were followed exactly as directed for plasma samples. The lipid metabolism parameters used were triglyceride (TG; A110-2–1), creatinine (CRE; C011-2–1), low-density lipoprotein cholesterol (LDL-C; A113-2–1), and total cholesterol (TCH; A111-2–1). The plasma antioxidant activity parameters included total antioxidant capacity (T-AOC; A015-1–2), SOD (A001-1–2), glutathione peroxidase (GSH-Px; A005-1–2), catalase (CAT; A007-1–1), and malondialdehyde (MDA; A003-1–2).

### Muscle amino acids

At the end of the experimental period, three goats from each group were randomly selected and slaughtered (*n* = 3 per group; 9 animals in total). The *longissimus dorsi* muscle was immediately separated, stored on dry ice, and then transferred to a −80°C freezer for subsequent AA and FA analysis. The AAs were detected according to the methods of GB 5009.124–2016 and Li et al. [26]. Briefly, samples were hydrolysed with acid (6 mol/L HCl solution), after that, one gram of muscle was weighed and added to 5 mL of a 6 mol/L hydrochloric acid solution, 2 drops of phenol were added, and the contents were mixed well. Next, the sample was hydrolysed at 110°C for 22 h, filtered into a 50 mL volume flask, and diluted with water to volume. Next, 1.0 mL was transferred into a 10 mL tube and then reduced pressure dried at 45°C, after which the sample was added to 1.0 mL of sodium citrate buffer (pH = 2.2) and mixed well. The mixture was filtered through a 0.22 µm membrane filter, and individual AAs were detected by using an AA analyser (Biochrom 30, Biochrom Ltd., Cambridge, United Kingdom).

### Muscle fatty acids

The FAs were detected according to the methods of GB 5009.168–2016 and Zou et al. [27]. Briefly, approximately 0.2 g of the muscle sample was added to 5 mL of sodium hydroxide methanol solution (2%), and 3.5 mL of boron trifluoride methanol solution (10%) was then added, mixed well, and heated for 2 min for methyl esterification. Next, the mixture was added to 2.5 mL of saturated sodium chloride solution and 5 mL of n-heptane and mixed well. The mixture was subsequently centrifuged at 3,000 × g for 10 min at 4°C, and the supernatant was collected for further FA analysis. The FA content was analysed via Agilent 7890A gas chromatography (GC; Agilent Technologies, USA) with a hydrogen flame ionisation detector and capillary column (Agilent DB-WAX, Agilent Technologies, USA; 30 m × 0.25 mm × 0.25 µm). The content of individual FAs was calculated based on its retention time, and the peak area was quantified.

### Statistical analysis

Each goat was considered the experimental unit. The sample size for growth performance, lipid metabolism and antioxidant activity parameters was eight (*n* = 8 per group; 24 animals in total); and the sample size for muscle AA and FA was three (*n* = 3 per group; 9 animals in total). The experimental design was completely randomized design in this study. In addition, before statistical analysis was conducted, the Levene’s analysis was tested the homogeneity of variance, and the Kolmogorov–Smirnov analysis was tested the normality of the distribution of variables. All raw data were calculated via Excel software, and then were analysed by a mixed procedure followed by the Tukey’s least significant difference post hoc comparison between the treatments. The treatments (CON, LF, and HF) were fixed effects, and experimental goats were random effects. A *P* < 0.05 indicated that the results were statistically significant, and the results were presented mean ± standard error of the mean (SEM).

## Results

### Growth performance

There were no significant differences (*P* > 0.05) in DMI, initial weight, final weight, BWC, or ADG among the three groups (Table 3). Compared with CON, FRRT supplementation reduced (*P* < 0.05) FCR.

**Table 3:** Effect of fermented *rosa roxburghii* tratt pomace on growth performance in goat.

Item	Treatment			SEM	*P*-value
	CON	LF	HF		
DMI, (g/d)	1044	1058	1041	13.905	0.653
Initial weight, (kg)	30.4	30.4	30.2	1.678	0.997
Final weight, (kg)	34.0	34.6	33.3	1.936	0.950
BWC, (kg)	3.17	3.52	3.50	0.789	0.960
ADG, (g/d)	58.6	65.2	64.8	14.619	0.960
FCR	17.8^a^	16.2^b^	16.1^b^	0.219	<0.001

^a-b^within the same row mean with different letters differ significantly (*P* < 0.05).

Values represent the means of eight replicates (*n* = 8). DMI: dry matter intake; BWC: body weight change; ADG: average daily gain; FCR: feed conversion ratio; SEM: standard error of the mean.

CON: goats were fed a basal diet; LF: goats were fed a basal diet supplemented with 7% fermented *rosa roxburghii* tratt pomace; HF: goats were fed a basal diet supplemented with 14% fermented *rosa roxburghii* tratt pomace.

### Plasma lipid metabolism

There were no significant differences (*P* > 0.05) in the plasma TG concentrations among the groups (Table 4). Compared with the those of the CON group, both the LF and HF treatment groups presented significantly higher (*P* < 0.05) CRE and TCH levels. In addition, LDL-C was numerically higher in the HF group (overall *P* = 0.069).

**Table 4:** Effect of fermented *rosa roxburghii* tratt pomace on plasma lipid metabolism in goat.

Item	Treatment			SEM	*P*-value
	CON	LF	HF		
TG, (mmol/L)	0.24	0.25	0.28	0.024	0.447
CRE, (μmol/L)	57.6^b^	75.6^a^	76.0^a^	2.508	0.003
LDL-C, (mmol/L)	0.32	0.57	0.71	0.103	0.069
TCH, (mmol/L)	0.73^b^	1.01^a^	0.99^a^	0.068	0.032

^a-b^within the same row mean with different letters differ significantly (*P* < 0.05).

Values represent the means of eight replicates (*n* = 8). TG: triglyceride; CRE: creatinine; LDL-C: low-density lipoprotein cholesterol; TCH: total cholesterol; SEM: standard error of the mean.

CON: goats were fed a basal diet; LF: goats were fed a basal diet supplemented with 7% fermented *rosa roxburghii* tratt pomace; HF: goats were fed a basal diet supplemented with 14% fermented *rosa roxburghii* tratt pomace.

### Plasma antioxidant activity

Dietary supplementation with FRRT had no significant (*P* > 0.05) effect on the plasma SOD and GSH-Px levels of the goats (Table 5). Compared with those in the CON group, the LF and HF treatment groups presented significantly (*P* < 0.05) increased plasma T-AOC and CAT levels. In addition, the MDA content tended to be lower in HF than CON (*P* = 0.067).

**Table 5:** Effect of fermented *rosa roxburghii* tratt pomace on plasma antioxidant activity in goat.

Items	Treatment			SEM	*P*-value
	CON	LF	HF		
T-AOC, (mmol/L)	0.56^b^	0.69^a^	0.69^a^	0.012	0.001
SOD, (U/mL)	100.7	101.0	101.3	0.294	0.366
GSH-Px, (U/mL)	168.8	173.1	168.8	6.386	0.863
CAT, (U/mL)	0.76^b^	1.20^a^	1.18^a^	0.092	0.013
MDA, (nmol/mL)	3.59	1.93	1.63	0.505	0.067

^a-b^within the same row mean with different letters differ significantly (*P* < 0.05).

Values represent the means of eight replicates (*n* = 8). T-AOC: total antioxidant capacity; SOD: superoxide dismutase; GSH-Px: glutathione peroxidase; CAT: catalase; MDA: malondialdehyde; SEM: standard error of the mean.

CON: goats were fed a basal diet; LF: goats were fed a basal diet supplemented with 7% fermented *rosa roxburghii* tratt pomace; HF: goats were fed a basal diet supplemented with 14% fermented *rosa roxburghii* tratt pomace.

### Muscle amino acids

Dietary FRRT supplementation had no effect (*P* > 0.05) on the muscle aspartate (Asp), glutamate (Glu), proline (Pro), alanine (Ala), valine (Val), methionine (Met), isoleucine (Ile), leucine (Leu), phenylalanine (Phe), histidine (His) or essential AA (EAA)/nonessential AA (NEAA) ratio (Table 6). However, the HF group presented greater (*P* < 0.05) threonine (Thr), serine (Ser), glycine (Gly), tyrosine (Tyr), lysine (Lys), arginine (Arg), essential AA (EAA), non-essential AA (NEAA), umami AA (FAA), and total AA (TAA) levels than the CON group.

**Table 6:** Effect of fermented *rosa roxburghii* tratt pomace on muscle amino acid in goat.

Item (g/100g)	Treatment			SEM	*P*-value
	CON	LF	HF		
Asp	1.57	1.57	1.71	0.037	0.060
Thr	0.74^b^	0.76^b^	0.82^a^	0.017	0.034
Ser	0.59^b^	0.60^b^	0.65^a^	0.012	0.027
Glu	2.43	2.50	2.68	0.060	0.061
Pro	0.65	0.63	0.68	0.012	0.067
Gly	0.74^b^	0.74^b^	0.80^a^	0.014	0.033
Ala	0.86	0.87	0.94	0.022	0.079
Val	0.71	0.72	0.76	0.014	0.110
Met	0.30	0.32	0.36	0.016	0.112
Ile	0.78	0.78	0.82	0.016	0.164
Leu	1.25	1.25	1.34	0.025	0.061
Tyr	0.50^b^	0.52^b^	0.55^a^	0.009	0.023
Phe	0.55	0.54	0.58	0.008	0.051
His	0.64	0.66	0.76	0.030	0.061
Lys	1.55^b^	1.59^b^	1.71^a^	0.034	0.031
Arg	1.02^b^	1.05^b^	1.14^a^	0.023	0.020
EAA	6.52^b^	6.62^b^	7.15^a^	0.132	0.030
NEAA	8.36^b^	8.48^b^	9.16^a^	0.168	0.031
EAA/NEAA (%)	78.1	78.0	78.1	0.815	0.997
FAA	6.61^b^	6.73^b^	7.27^a^	0.142	0.036
TAA	14.9^b^	15.1^b^	16.3^a^	0.304	0.031

^a-b^within the same row mean with different letters differ significantly (*P* < 0.05).

EAA: Thr + Val + Met + Ile + Leu + Phe + His + Lys; NEAA: Asp + Ser + Glu + Pro + Gly + Ala + Tyr + Arg; FAA: Asp + Glu + Gly + Ala + Arg.

EAA: essential amino acid; NEAA: non-essential amino acid; FAA: flavor amino acid; TAA: total amino acid. Thr: threonine; Val: valine; Met: methionine; Ile: isoleucine; Leu: leucine; Phe: phenylalanine; His: histidine; Lys: lysine; Asp: aspartate; Ser: serine; Glu: glutamate; Pro: proline; Gly: glycine; Ala: alanine; Tyr: tyrosine; Arg: arginine; SEM: standard error of the mean.

CON: goats were fed a basal diet; LF: goats were fed a basal diet supplemented with 7% fermented *rosa roxburghii* tratt pomace; HF: goats were fed a basal diet supplemented with 14% fermented *rosa roxburghii* tratt pomace.

### Muscle fatty acids

Dietary FRRT supplementation had no effect (*P* > 0.05) on the concentrations of C14:0, C16:0, C17:0, C18:0, saturated FA (SFA), C16:1, C17:1, C18:1n9c, C22:1n9, MUFA, C18:2 n-6 (cis 9,12), C18:3n3, C20:3n6, C20:5n3, C22:6n3, or PUFA (Table 7) C15:0 was not detected in the HF treatment, and C20:0 and C20:1 were not detected in either FRRT treatment. The C15:0 content in the CON group was greater (*P* < 0.05) than that in the LF treatment group. In contrast, the level of C20:4n6 in the LF treatment group was significantly higher (*P* < 0.05) than that in the CON group.

**Table 7:** Effect of fermented *rosa roxburghii* tratt pomace on muscle fatty acid in goat.

Item (%)	Treatment			SEM	*P*-value
	CON	LF	HF		
C14:0	0.99	0.89	0.90	0.167	0.892
C15:0	0.86^a^	0.65^b^	nd	0.024	0.004
C16:0	22.1	18.2	20.4	1.787	0.368
C17:0	2.03	1.36	1.32	0.245	0.152
C18:0	16.2	12.2	11.2	1.581	0.143
C20:0	0.31	nd	nd	–	–
SFA	42.4	33.3	33.9	3.055	0.139
C16:1	2.11	1.69	2.73	0.316	0.145
C17:1	0.94	0.81	0.93	0.103	0.661
C18:1n9c	27.5	29.0	31.0	3.014	0.727
C20:1	0.60	nd	nd	–	–
C22:1n9	1.65	1.95	1.65	0.441	0.857
MUFA	32.8	33.4	36.3	2.880	0.678
C18:2 n-6 (cis 9,12)	10.5	13.1	14.3	1.287	0.190
C18:3n3	2.31	2.71	1.78	0.434	0.375
C20:3n6	1.12	1.55	1.14	0.302	0.559
C20:4n6	7.29^b^	12.2^a^	10.0^ab^	0.898	0.023
C20:5n3	2.37	2.00	1.48	0.269	0.138
C22:6n3	1.23	1.70	1.17	0.194	0.188
PUFA	24.8	33.2	29.9	2.755	0.177

nd = not detected. For not detected data, all not detected data were excluded and other data were conducted further analysis. ^a-b^within the same row mean with different letters differ significantly (*P* < 0.05).

SFA: saturated fatty acids; MUFA: monounsaturated fatty acids; PUFA: polyunsaturated fatty acid; SEM: standard error of the mean.

CON: goats were fed a basal diet; LF: goats were fed a basal diet supplemented with 7% fermented *rosa roxburghii* tratt pomace; HF: goats were fed a basal diet supplemented with 14% fermented *rosa roxburghii* tratt pomace.

## Discussion

The RRT contains high levels of vitamins and polyphenol components, which show potent *in vitro* scavenging activity against hydroxyl and 2,2-diphenyl-1-picrylhydrazyl (DPPH) radicals, enhancing animal immune function [16]. In the current study, the reduction in FCR might be attributed to two non-exclusive factors: (1) FRRT contains high concentrations of polyphenol compounds (Table 1), which could increase the antioxidant capacity and improve immunity in goats, thus reducing the FCR [28,29]; and (2) FRRT contains other bioactive ingredients, such flavonoids and essential oils, which could support the development of beneficial rumen microbes [30,31]. Our results were consistent with those of Bahrampour et al. [32], who demonstrated that the consumption of grape byproducts decreased FCR in lambs. In brief, the finding of this study suggested that FRRT was a source of unconventional feed for ruminants because FRRT did not negatively affect production performance in meat goats, whereas it could decrease the FCR value.

Polyphenols are a class of compounds with aromatic rings modified by multiple hydroxyl groups, and most polyphenols and their derivatives contain both sugars and other residues [33]. Previous studies have shown that a high sugar diet can stimulate insulin secretion, thereby promoting fat synthesis and deposition in animals [34,35]. The results revealed that dietary addition of FRRT could increase plasma lipid metabolism parameters, such as CRE and TCH in goats. It is hypothesized that this pattern (specifically, the increase in TCH and CRE in both FRRT groups and the rise in LDL-C only in the HF group) may be partly attributable to the sugar moieties within FRRT polyphenols. These components could potentially influence postprandial insulin secretion and subsequent hepatic lipid metabolism, thereby promoting lipid deposition and improving lipid metabolism parameters. However, this conclusion needs to be further confirmed in the future. In contrast, a previous study showed that polyphenols decreased plasma total cholesterol via the suppression of intestinal lipid metabolism in animals [36]. A pertinent example is the work of Ahmed et al. [12], who reported that the addition of green tea byproducts to the diet of goats caused a linear decrease in plasma glucose and cholesterol and a quadratic decrease in urea nitrogen concentration. The reason for these differences might as followed: (1) different composition and proportions of polyphenol compounds present in the green tea byproducts versus FRRT; and (2) differences in polyphenol profiles between sources can lead to very different metabolic outcomes. In addition to the difference in polyphenol composition itself, dose effects, basal diet interactions, species specificity, and FRRT-specific fermentation-derived metabolites may all lead to very different metabolic results; this study did not consider the effect of different FRRT on lipid metabolism, which was a potential limitation in this study.

The RRP contains a variety of active chemicals that can be utilised as good sources of antioxidants [37,38]. Additionally, a polyphenol-rich diet can protect cells from free radical damage by either increasing the levels of endogenous antioxidant enzymes [39,40] or altering the expression of the enzymatic antioxidant system [41]. The current study demonstrated that FRRT could increase T-AOC and CAT concentrations and the MDA content tended to be lower in HF than CON, this possible reason may as followed: (1) FRRT has an abundance of polyphenolic compounds, and polyphenol compounds from FRRT enhance the scavenging effect of 1,1-diphenyl-2-picryl-hydrazyl radical (DPPH·), hydroxyl radical (·OH), and superoxide radical (O₂ ⁻ ·). Specifically, this translated to enhanced CAT activity and total antioxidant capacity (T-AOC), with no significant changes in SOD or GSH-Px activities [42]; and (2) bioactive compounds (e.g., polyphenol, sugar) improved the antioxidant signaling pathway, and up-regulated some antioxidant gene expressions, and then enhanced antioxidant enzymes in ruminants [43]. Consistent with our findings, Sharifi et al. [44] reported that the addition of palm *(Phoenix dactylifera L*.) seeds increased the blood antioxidant activity of dairy goats.

Polyphenols increase the abundance of several AA transporters, such as SLC7A1, SLC7A2, SLC7A7, and SLC1A2, improve nitrogen metabolism and subsequently increase muscle AA content in animals [45]. Moreover, polyphenols can be delivered to lower parts of the gastrointestinal tract via polyphenol–protein interactions in animals [46]. Specifically, some high-protein agro-industry byproducts contain many more phenols, including chlorogenic and caffeic acids, which can combine with proteins and thus affect the AA content [47]. The present study demonstrated that feeding FRRT increased the muscle AA content mainly in the HF group goats, possibly because the polyphenols in FRRT forming complexes with dietary proteins. This interaction could potentially shield the protein from extensive ruminal degradation, facilitating its passage to the small intestine for more efficient absorption and post-absorptive nitrogen metabolism [48], and then improved AA content in goats. However, the relevant mechanism is still unclear, further studies are necessary to determine the rumen bypass rate and clarify the specific interaction mechanism. Consistent with our results, Wang et al. [49] reported that adding oat supplements to the diet can increase the AA level of muscle of the small-tailed Han sheep and improve meat quality. However, this study only evaluated male Guizhou black goats; breed and sex effects were not tested. The muscle analyses used n = 3 per group, which limits power.

The addition of antioxidants could protect against FA peroxidation by decreasing the toxic effects of unsaturated FA (UFAs) on the rumen microbiota [50]. Serra et al. [10] reported that the inclusion of a polyphenol-rich diet in ruminants reduced the biohydrogenation of PUFA and thus increased the PUFA concentration. Additionally, polyphenols can prevent PUFAs in goat meat from oxidising instead of more stable SFAs [51,52]. Thus, the results of this study showed that dietary addition of FRRT could improve the *longissimus dorsi* muscle C20:4n6 content, possibly because bioactive compounds from FRRT could exert their antioxidant function in the protection of PUFA in feed and muscle, reduce the biohydrogenation of PUFA in goats, and increase the muscle C20:4n6 content [53]. The observations agree with the findings of Natalello et al. [54], who reported that the inclusion of whole pomegranate byproduct could increase muscle antioxidants and then increase PUFA concentrations in lambs. However, the effect of FRRT on sensory indicators, such as meat flavor and juiciness need to be further evaluated.

## Conclusion

The results of the present study indicate that FRRT is an excellent unconventional feed for ruminants because it contains an abundance of polyphenol compounds. Dietary supplementation with FRRT could reduce FCR and improve plasma antioxidant status and muscle AA and FA contents in meat goats. RRT is widely available in Guizhou but its pomace is wet (>80% moisture) and poorly storable; fermentation into FRRT offers a practical route to valorise this by product as a functional roughage ingredient. Future work should address supply chain consistency and cost-benefit in commercial settings.

## Supporting information

S1 Table Polyphenol compounds of pure fermented *rosa roxburghii* tratt pomace. (DOCX)

S2 Table The ingredients and chemical composition of experimental diets (dry matter basis). (DOCX)
