# Supplementary material for: Effects of fermented Rosa roxburghii Tratt pomace on growth performance, lipid metabolism, antioxidant activity, and amino and fatty acid profile in goats
Source: PLoS One. 2026 Feb 3;21(2):e0342308. doi: 10.1371/journal.pone.0342308 (PMC12867216; doi:10.1371/journal.pone.0342308)
Supplement: S1 Table — (DOCX) [file pone.0342308.s001.docx]

**S1 Table. Polyphenol compounds of pure fermented *rosa roxburghii* tratt pomace.**

| Item | Polyphenol compounds (ng/mg of dry matter) |
| --- | --- |
| Gallic acid | 44.7±5.03 |
| 3,4-Dihydroxybenzoic acid | 10.8±1.41 |
| Protocatechualdehyde | 0.83±0.09 |
| 4-Hydroxybenzoic acid | 5.02±0.32 |
| Phthalic acid | 2.25±0.18 |
| Catechin | 7.55±0.63 |
| Vanillic acid | 8.58±0.44 |
| Caffeic acid | 4.22±0.81 |
| Syringic acid | 4.46±0.36 |
| Dihydromyricetin | 0.08±0.02 |
| Vanillin | 1.42±0.09 |
| p-Hydroxycinnamic Acid | 6.46±0.39 |
| Syringaldehyde | 0.63±0.06 |
| Rutin | 0.12±0.02 |
| Vitexin | 0.07±0.01 |
| Trans-Ferulic acid | 20.3±2.21 |
| Salicylic acid | 0.95±0.03 |
| Sinapic Acid | 0.62±0.04 |
| Quercetin 3-β-D-glucoside | 0.52±0.11 |
| (+)-Dihydroquercetin | 2.41±0.07 |
| Genistin | 0.08±0.02 |
| Benzoic acid | 3.38±0.20 |
| Kaempferol-3-O-glucoside | 1.62±0.11 |
| (+)-Dihydrokaempferol | 0.89±0.02 |
| Resveratrol | 0.05±0.00 |
| Luteolin | 0.24±0.01 |
| Quercetin | 24.0±1.48 |
| Hydrocinnamic acid | 0.96±0.11 |
| Trans-Cinnamic acid | 0.99±0.10 |
| Phloretin | 0.01±0.00 |
| Apigenin | 0.06±0.00 |
| Naringenin | 0.87±0.05 |
| Kaempferol | 11.3±1.06 |
| Isorhamnetin | 0.44±0.02 |
| Gossypol | 0.74±0.12 |

Values represent the means of six replicates (n = 6). mean ± standard deviation.
