# Supplementary material for: Effects of fermented Rosa roxburghii Tratt pomace on growth performance, lipid metabolism, antioxidant activity, and amino and fatty acid profile in goats
Source: PLoS One. 2026 Feb 3;21(2):e0342308. doi: 10.1371/journal.pone.0342308 (PMC12867216; doi:10.1371/journal.pone.0342308)
Supplement: S2 Table — (DOCX) [file pone.0342308.s002.docx]

**S2 Table. The ingredients and chemical composition of experimental diets (dry matter basis).**

| Item | CON | LF | HF |
| --- | --- | --- | --- |
| Oat grass | 53.0 | 46.0 | 39.0 |
| Hybrid giant napier | 7.00 | 7.00 | 7.00 |
| Fermented *rosa roxburghii* tratt pomace | 0.00 | 7.00 | 14.0 |
| Corn | 21.4 | 22.2 | 23.1 |
| Soybean meal | 14.2 | 13.3 | 12.4 |
| Salt | 0.50 | 0.50 | 0.50 |
| Sodium bicarbonate | 0.60 | 0.60 | 0.60 |
| Compound-premix^*^ | 3.40 | 3.40 | 3.40 |
| Chemical composition, % |  |  |  |
| DM | 90.0 | 90.2 | 89.7 |
| CP | 14.5 | 14.5 | 14.6 |
| EE | 3.94 | 3.58 | 3.23 |
| NDF | 59.2 | 55.6 | 51.7 |
| ADF | 31.8 | 30.5 | 29.7 |
| Ash | 7.70 | 7.09 | 6.62 |

Values represent the means of three replicates (*n* = 3). DM: dry matter; CP: crude protein; EE: ether extract; NDF: neutral detergent fiber; ADF: acid detergent fiber.

^*^The compound-premix contains a vitamin and mineral premix with the following composition per kg: vitamin A, 150 KIU; vitamin D3 80 KIU, vitamin E, 500 IU, Cu 300 mg, Fe 1500 mg, Zn 1500 mg, Mn 1500 mg, I 30 mg, Co 10 mg, Se 5 mg.

CON: goats were fed a basal diet; LF: goats were fed a basal diet supplemented with 7% fermented *rosa roxburghii* tratt pomace; HF: goats were fed a basal diet supplemented with 14% fermented *rosa roxburghii* tratt pomace.
